# Elemental and Isotopic Characterization of Tobacco from Umbria

**DOI:** 10.3390/metabo11030186

**Published:** 2021-03-22

**Authors:** Luana Bontempo, Daniela Bertoldi, Pietro Franceschi, Fabio Rossi, Roberto Larcher

**Affiliations:** 1 Research and Innovation Centre, Fondazione Edmund Mach (FEM), Via E. Mach 1, 38098 San Michele all’Adige, Italy; pietro.franceschi@fmach.it; 2 Technology Transfer Centre, Experiment and Technological Services Department, Fondazione Edmund Mach (FEM), Via E. Mach 1, 38098 San Michele all’Adige, Italy; daniela.bertoldi@fmach.it (D.B.); roberto.larcher@fmach.it (R.L.); 3 Fattoria Autonoma Tabacchi Soc. Coop Agricola, Via G. Oberdan, 06012 Città di Castello, Italy; fabio.rossi@fattoriatabacchi.it

**Keywords:** stable isotope ratios, elemental composition, tobacco, geographical origin

## Abstract

Umbrian tobacco of the Virginia Bright variety is one of the most appreciated tobaccos in Europe, and one characterized by an excellent yield. In recent years, the Umbria region and local producers have invested in introducing novel practices (for production and processing) focused on environmental, social, and economic sustainability. Due to this, tobacco from Umbria is a leading commodity in the global tobacco industry, and it claims a high economic value. The aim of this study is then to assess if elemental and isotopic compositions can be used to protect the quality and geographical traceability of this particular tobacco. For the first time the characteristic value ranges of the stable isotope ratios of the bio-elements as a whole (δ^2^H, δ^13^C, δ^15^N, δ^18^O, and δ^34^S) and of the concentration of 56 macro- and micro-elements are now available, determined in Virginia Bright tobacco produced in two different areas of Italy (Umbria and Veneto), and from other worldwide geographical regions. The ranges of variability of elements and stable isotope ratios had slightly different results, according to the three geographical origins considered. In particular, Umbria samples presented significantly lower content of metals potentially dangerous for human health. The results of this first exploratory work highlight the possibility of characterizing tobacco from Umbria, and suggest widening the scope of the survey throughout Italy and foreign regions, in order to be used to describe the geographical origin of tobacco in general and verify the origin of the products on the market.

## 1. Introduction

Tobacco is the common name of several plants of the *Nicotiana* genus (Solanaceae family) and the general term for any product prepared from the cured leaves of the tobacco plant. Besides its hedonistic use, tobacco plays an important role in other sectors, and has been increasingly investigated in the last few years [1]. Its alkaloid nicotine is widely used in agriculture as a contact insecticide [2], refined oil of tobacco seeds is used as edible oil [3,4] and has been proposed for the production of biodiesel [5], and tobacco seeds cake are used as feed for animals [6]. Last but not least, tobacco has been investigated for medical applications, and plays an important role in the advancement of plant biotechnology, as it is an extremely versatile system for genetic manipulation and tissue culture research [6,7,8,9,10,11].

Tobacco is currently grown in 12 European countries. The main producers are Italy, Spain, Poland, Greece, Croatia, France, Hungary, and Bulgaria, accounting for 99% of the European Union’s (EU’s) tobacco production [12]. The European Union produces around 200,000 tonnes of tobacco per year (data of 2019 from FAOSTAT—Statistic by The Food and Agriculture Organization, accessed on 4 March 2021). The most popular tobacco variety in the world is the Virginia Bright flue-cured tobacco, because of its unique—and easily replicated—cultivation and curing methods, and it is a leading commodity in the global tobacco industry. In particular, flue-cured Virginia varieties account for 71% of output, while light-air-cured Burley varieties are 16%, sun-cured or oriental 7%, and other varieties (dark-air-cured and fire-cured) 6% [12]. Italy is the leading producer of raw tobacco in the European Union, and among the 10 leading tobacco-producing countries worldwide (data from the Italian Ministry of Agriculture and the European Commission EC [12,13]). In Italy, 97% of tobacco is grown in only four regions: Campania, Umbria, Veneto, and Tuscany. The aromatic Virginia Bright, considered the best Italian and European flue-cured tobacco [14], is grown in the Veneto region, whereas the Virginia Filler variety, one of the most appreciated tobaccos in Europe and one characterized by an excellent yield, comes from the Umbria region. In the last few years, in general but in particular in Umbria, producers have tended to offer a high-quality product with a transparent and short supply chain, cultivated with the least possible impact on the surrounding environment, in compliance with the good agricultural practices proposed by CORESTA (Cooperation Centre for Scientific Research Relative to Tobacco) and with the agri-environmental measures defined at the local and national and international levels (social responsibility). The Umbria region and local producers, in particular, have worked side-by-side in order to reduce the impact of this cultivation on the environment and on the health of workers, as well as the health of the entire communities living in the area, and produce a product of high quality. In order to offer products of higher quality, their chemical characterization is of paramount importance, in particular to assess and quantify undesirable and harmful byproducts (e.g., toxic elements) directly from the *Nicotiana tabacum* leaves. Even if many studies have been conducted to characterize *Nicotiana tabacum* from the genetic and molecular points of view (e.g., [15,16]), to the best of our knowledge few papers have focused on the elemental characterization of *Nicotiana tabacum* leaves and plants [17,18,19,20]. To date, the majority of the chemical investigations have been limited to the assessment of the presence of toxic elements that can be inhaled or absorbed through cigarette smoke [21,22,23]. 

In order to support the quality assessment, the chemical characterization has to be coupled with analytical techniques that enable the characterization and traceability of the provenance of tobacco, and that can objectively guarantee it. In this area, however, up to now, very few studies focused on the development of analytical methods able to trace the geographical origin of tobacco [24,25]. Stable isotope ratios of bio-elements, as well as elemental composition, have been successfully used in the last 30 years to trace and verify the origin of several foodstuffs [26,27]. Isotopic fractionation occurs during physical and chemical processes, as well as along metabolic pathways, and for this reason, the different isotopic ratios vary according to photosynthetic and nitrogen cycles, pedological characteristics of soils, agricultural practices, and geographical origin. The elemental composition is mainly affected by soil geology and pedological characteristics, but can be also a consequence of contamination during processing. The combination of stable isotope analysis and elemental composition is expected to be a powerful tool for the assessment of the geographical origin, as has been already widely demonstrated, for example, in food traceability [28,29]. In the particular case of tobacco, only a few papers have been published on the application of stable isotope ratio and trace element profiles to the tracing of its geographical origin. As far as stable isotope ratios are concerned, only two studies were published on the geographical traceability of tobacco, to the best of our knowledge. In 1997, Jamin and colleagues [25] published a paper on the multi-element and multi-site isotopic analysis of purified nicotine extracted from tobacco leaves, concluding that the carbon and nitrogen isotopic composition (^13^C/^12^C, ^15^N/^14^N) and the site-specific deuterium content of nicotine can provide useful information on the geographical origin of tobacco. Similarly, Binette and colleagues [30] found that the combination of ^2^H/^1^H and ^15^N/^14^N ratios can be useful for identifying foreign counterfeits of Canadian cigarettes. To date, the elemental composition of tobacco has been investigated mainly to assess the presence of toxic elements that can be inhaled or absorbed through cigarette smoke [21,22,23]. Only one paper has focused on the use of elements to differentiate between tobaccos from different origins. Kazi and colleagues [21], indeed, have determined the content in Al, As, Cd, Ni, and Pb of filler tobacco of different local and imported branded cigarettes sold in Pakistan. Furthermore, Judd and Swami [31] found different values of the isotopic ratios of lead for cigarette tobaccos grown in different regions.

To date, the stable isotope ratios of H, C, N, O, and S, and the elemental composition of *Nicotiana tabacum* have never been extensively explored for their characterization and the verification of their origin. Therefore, the aim of this study is to fill in this gap and start exploring and determining the characteristic value ranges for elements and the stable isotope ratios of the main cultivated tobacco variety (Virginia Bright). In particular, the investigation focuses on Virginia Bright from Umbria, where it has been cultivated for centuries, and where the cultivation is strictly linked to these territories. Furthermore, in recent years in this area, tobacco producers have changed their way of manufacturing, giving particular attention to environmental, social, and economic sustainability along the entire production chain, from cultivation to processing, in order to obtain products of higher quality. The aim was then to assess if the combination of elemental and isotopic compositions, as already successfully applied in the field of food traceability, can be used to protect the quality and geographical traceability of tobacco from Umbria. In particular, this paper contributes to the creation of the very first isotopic and elemental database, also including toxic elements, for Umbrian Virginia Bright tobacco, comparing the obtained data with data of Virginia Bright samples coming from other geographical regions.

## 2. Results and Discussion

### 2.1. General Description of the Isotopic and Elemental Content, According to the Geographical Origin 

The isotopic and elemental data variability of tobacco from Umbria, Veneto, and other geographical origins are reported in the Supplementary Materials (Table S1). Despite a general overlapping of isotopic and elemental data for samples from different geographical origin, it is worth noting that some geographical areas are characterized by a peculiar range of values that is different from that of the samples from the other countries (Table S1). In particular, China presented lower values of δ^18^O and basically higher values of Be, Pd, Cd, In, and Bi, whereas the African samples showed basically higher values of Rb, Ba, and La. The Argentinian samples presented a higher value of Li, while Brazil had higher values of Mg and Rb. δ^34^S was higher for the United States samples, and lower for the Umbria samples. Hungary showed basically higher values of Mn. Such data were checked using the non-parametric Kruskal–Wallis approach (*p* < 0.05) to detect statistically significant differences according to the geographical origin. It is interesting to note that, as regards the specific characteristics of tobacco with different geographical origin, despite the geographical proximity of Umbria and Veneto, the Umbrian samples presented statistically different values of δ^2^H, δ^13^C, δ^18^O, δ^34^S, Li, Mo, and Bi (*p* < 0.05; median values −89‰ versus −108‰, −26.5‰ versus −28.3‰, 27.3‰ versus 24.3‰, 1.1‰ versus 6.4‰, 12.9 mg/kg versus 2.4 mg/kg, 0.37 mg/kg versus 1.22 mg/kg, and 1.8 µg/kg versus 5.7 µg/kg) from Veneto. The different δ^2^H, δ^13^C, and δ^18^O values can be explained by the latitude effect. Indeed, mean latitude in Umbria is 43°, whereas Veneto is at a higher latitude of around 45.5°. According to Dansgaard [32], the latitude affects the isotopic values of precipitation water, and therefore of the plant material, which shows a decrease of the δ^2^H and δ^18^O values as latitude increases. A similar effect, albeit smaller compared to that of δ^2^H and δ^18^O, occurs also in δ^13^C, and is related to the water stress and light irradiance conditions. Indeed, in warmer climate regions, plants tend to close their stomata under conditions of water stress and high light irradiance, which results in an enrichment in δ^13^C values by a few ‰ [33].

As regards δ^34^S, Li, Mo, and Bi, the content differences by geographical origin can be related to the different geological origin of the areas considered, as well as to the pedological characteristics of the soil and the proximity to the sea [29,34].

It is noteworthy that the concentration of rare earth elements (RREs) in the Umbria samples is generally lower than that of the products from other locations, probably reflecting the different distributions of RREs in the growth soils. The concentrations of RREs are in agreement with the values already reported in other studies on vegetal matrices [35,36], confirming that the content of light REEs (La, Ce, Pr, Nd, and Sm) is significantly higher than that of the heavy REEs (Gd, Dy, Ho, Tm, and Yb). In particular, lanthanides, as well as Y, are significantly different (*p* < 0.05) between the Umbrian samples and those from Poland and Africa. The results of previous works suggest the usefulness of these parameters in food authentication [36,37].

The content of Be, Na, Mg, Cr, Ni, Ga is statistically different between Umbria and Spain, Africa, Hungary, Greece, and Poland. Ge, As, Rb, and Ag resulted as statistically different (*p* < 0.05) in Umbria compared to Poland, Veneto compared to Spain, Greece compared to Africa, and Bulgaria compared to Hungary. Furthermore, the Umbria samples were statistically different (*p* < 0.05) on the basis of the content in Tl (from those of Africa), Th and U (from those of Spain and Poland), and Pb and Te (from those of Bulgaria and Poland).

### 2.2. Characterization of Umbrian Tobacco Versus Tobacco from Veneto and Other Geographical Origins 

**Figure 1:**
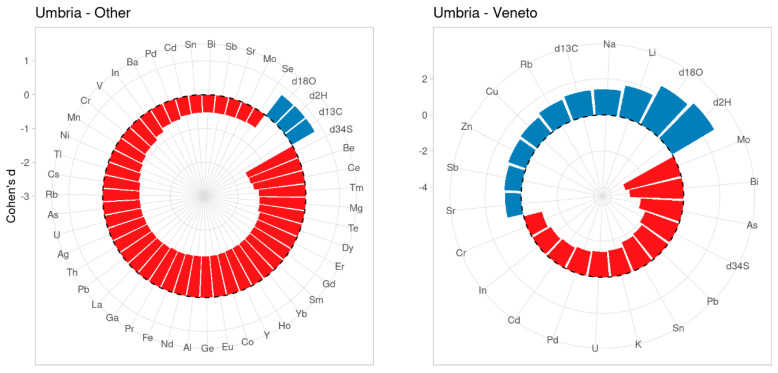
Plots of Cohen’s *d* effect size (standardized mean difference (SMD)) for tobacco leaves from Umbria, Veneto, and other geographical origin.

Figure 1 summarizes the results of the univariate statistical analysis in terms of Cohen’s *d* for pairwise comparison between the samples belonging to Umbria, Veneto, and other geographical origin. The full set of analytical data for the samples collected in Veneto, Umbria, and elsewhere was considered. The plots show the variables that showed significant results from the Wilcoxon non parametric test, and make it possible to check the strength of the difference between the values for the three groups and of the relationship between variables (blue means a positive direction, red a negative direction). Further details about the distribution of the data within each group are shown in the Supplementary Materials (Figure S1). 

According to Figure 1, it is immediately clear that the effect size and thus the strength of the difference between groups, even if limited to a smaller number of variables, is much larger in the comparison between Umbria and Veneto than between Umbria versus “other”. This is related to the extreme isotopic and elemental variability of the samples from the “other” group. Furthermore, the greater effects in the comparison between Umbria and “other” go in a negative direction only, whereas in the comparison between Umbria and Veneto, the effect is equally distributed between a negative and positive way. This is due to the fact that the majority of the elements in the Umbria samples are present in a lower concentration than in the “other” samples. 

In particular, in the comparison between Umbrian and Veneto samples, the highest positive effect (highest Cohen’s *d* values) was found for δ^2^H, δ^18^O, Li, Na, and δ^13^C (in decreasing order) and for Mo and Bi in the opposite direction. It is interesting to note that the main differences between the Umbria and Veneto samples pertain to the isotopes and the content of elements like Na and Li, which are alkali metals already used for the geographical traceability of food products [38,39]. In the comparison between Umbrian and “other” samples, the highest effect was found for δ^34^S, Be, Ce, Tm, Mg, and Te, in an inverse relationship. It is noteworthy that many of the elements having a higher impact on the differences between the Umbria and “other” samples belong to the rare earths. 

More generally, Umbrian tobacco is distinguishable from tobacco with other geographical origins (both the Veneto and “other” groups) by the value of the isotopic ratios of δ^2^H, δ^13^C, and δ^18^O, which are significantly higher, probably in relation to the particular climatic and geographic conditions of the area (Figure S1). Indeed, Umbria is located at a lower latitude (around 43° N) and is characterized by a lower amount of total rainfall and a higher average temperature during the year (around 800 mm/year and 15 °C) than Veneto (around 45° N, 1000 mm rainfall/year, and 13 °C). On the other hand, it has lower values of the isotopic ratio δ^34^S, which is related to the soil characteristics. Again, in relation to the characteristics of the soil, Umbrian tobacco has significantly lower contents of Al, Be, Mg, V, Cr, Mn, Fe, Co, Ni, Ga, Ge, As, Rb, Y, Pd, Ag, Cd, In, Te, Cs, La, Ce, Pr, Nd, Sm, Eu, Gd, Dy, Ho, Er, Tm, Yb, Tl, Pb, Bi, Th, U, Se, Mo, Sr, Sb, Sn, and Ba compared to the “other” group. On the other hand, it has higher content of Na. Umbrian tobacco has similar and not significantly different contents (*p* > 0.05) in all the elements, except for As, Mo, Pd, Ag, Sn, Pb, Bi, U, Cr, In, Cd, K (lower values in Umbria), Li, Na, Cu, Zn, Sr, Sb, and Rb (higher values in Umbria) when compared to Veneto tobacco. Metals and elements are acquired by the growing tobacco plant from soil, fertilizers, and industrial pollution. In addition, metals and elements also derive from tobacco curing treatments and processing (World Health Organization (WHO) Technical Report, 2012). Therefore, the amounts of metals and elements in tobacco products vary widely, depending on the geographical location in which the tobacco leaf is grown, as well as on the treatments administered during the processing (WHO Technical Report, 2012). In particular, it should be noted that the content in elements that are potentially dangerous for human health (e.g., As, Cd, Pb, Sn, Be, Cr, and Tl) is significantly lower in Umbrian tobacco. Furthermore, Umbrian data for Al, As, Ba, Be, Cd, Co, Cr, Fe, Mn, and Pb were lower overall, whereas Cu and Ni contents were quite similar to data reported by the World Health Organization for cigarette tobacco (WHO Technical Report, 2012), therefore indicating a high-quality product in this respect. The same situation was recorded for the Veneto samples, whereas in the case of the “other” group, the content in these elements varies much more, as expected due to the different origins. This point is particularly important, as the World Health Organization recommends regulatory authorities to test cured tobacco in particular for its content in As, Cd, Pb, and Ni, in order to limit human exposure to these particularly toxic elements.

**Figure 2:**
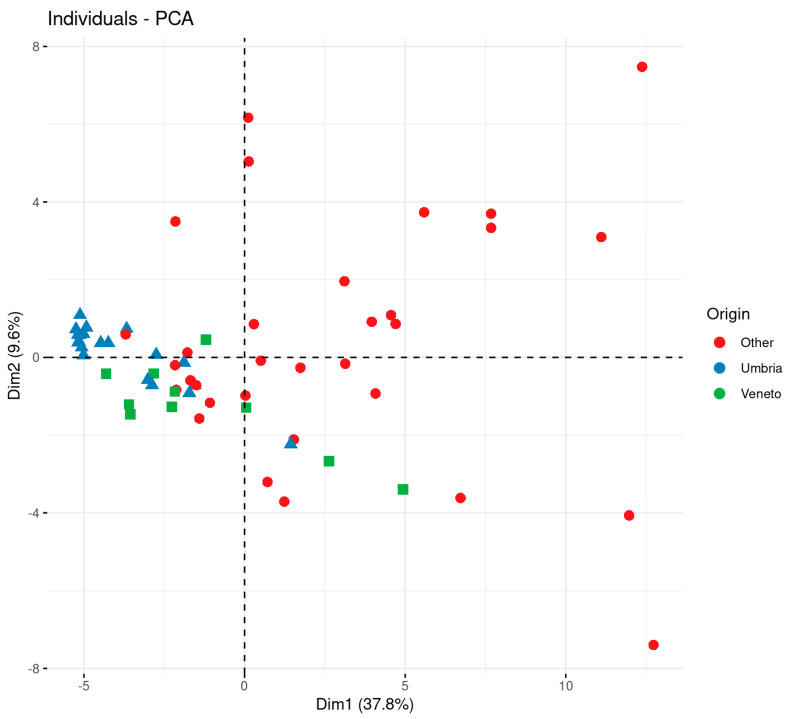
Score plot of principal component (PC)1 and PC2 for isotopic and elemental values of tobacco samples, according to their geographical origin (Umbria region versus other origins).

Due to the encouraging results of the univariate analysis, a multivariate, unsupervised statistical approach was applied by combining the 59 variables together, in order to highlight the factors that contribute to the differentiation between the Umbrian tobacco and tobacco from Veneto and other geographical origins. Principal component analysis (PCA) was performed on data, and the relative score plot is shown in Figure 2. 

**Figure 3:**
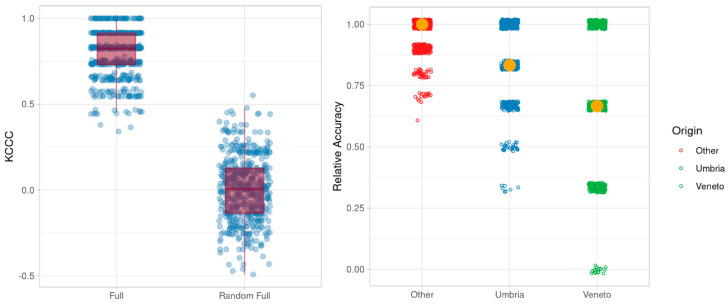
Ability of the developed model to classify origin correctly.

The plot shows that the three classes of samples are partially separated in a projection, which accounts for almost 50% of the variance (two principal components (PCs), with 38% variance explained for the first PC and 10% for the second PC). The isotopic and elemental composition of the Umbrian (blue triangles) and Veneto samples (green squares) are generally different from those of most samples of “other” origins (red dots). In particular, the majority of the samples of “other” origin fall into the region of the PCA, with positive values of PC1. The distribution of the “other” origin samples is much more spread than that of the Umbria and Veneto samples, confirming a more varied isotopic and elemental composition, due to the different geographical provenances. In order to identify the most influential variables that are driving the separation in the multivariate space, the random forest (RF) approach was applied. The outcomes of this analysis were validated with the “random region” scheme described in the Material and Methods section. The overall efficiency of the classifier is shown in Figure 3 in terms of kCCC (k Category Correlation Coefficient). The plot clearly indicates that all the classifiers with the correct class labels performed markedly better than the randomly assigned labels, showing that isotopic and elemental data can be used to distinguish Umbrian and non-Umbrian tobacco samples with good accuracy. This means that the results of the chemical analysis of a representative database of reference samples could potentially be used within an RF model to verify the origin of Umbrian tobacco in commercial samples as well. In terms of class-specific accuracy, the model was able to classify almost completely the samples belonging to the “other” class (median relative accuracy = 100%), but a good performance was also obtained for Umbrian tobacco (median relative accuracy 80%) (Figure 3). 

**Figure 4:**
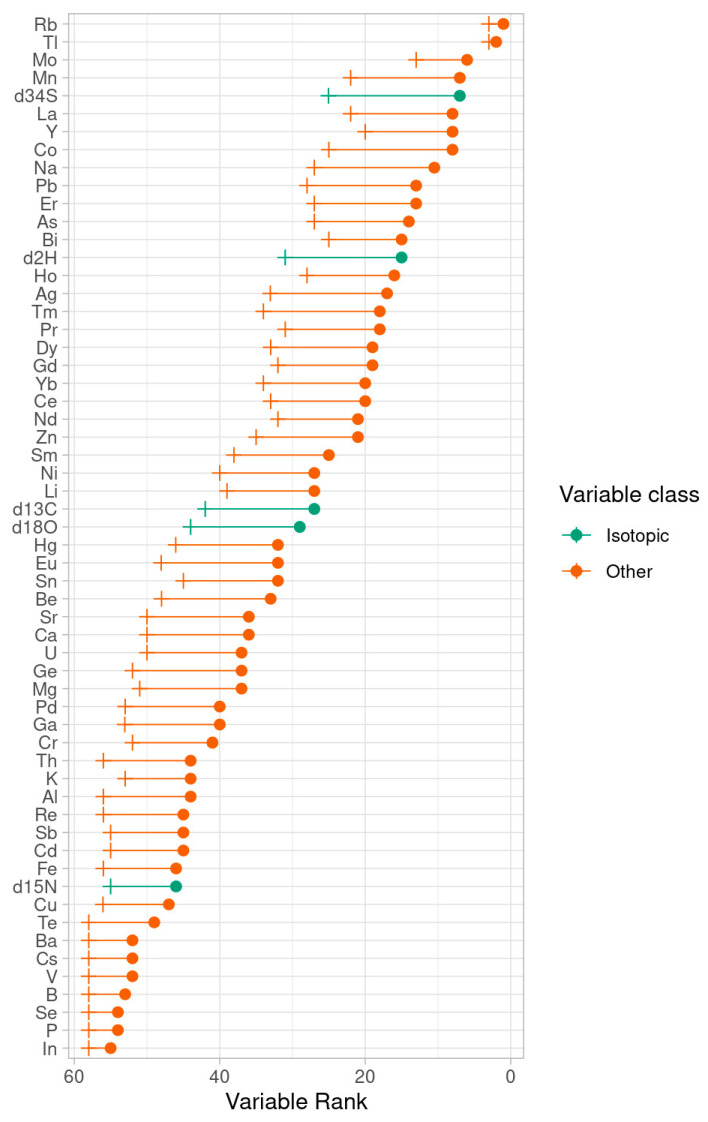
Random forest analysis of the isotopic and elemental compositions of tobacco leaves from Umbria, Veneto and other geographical origins: variable importance.

The variables with the most predictive power are reported in Figure 4. In the plot, each variable is characterized by its median rank across the set of validation runs (dot) and by the 0.95 quantile of its position in the variable importance list (cross). Two higher values of both parameters indicate that a variable is always important in the different classification models.

In particular, Rb and Tl in primis, followed by Mo, Mn, δ^34^S, La, Y, Co, Na, Pb, Er, As, Bi, and δ^2^H, showed a consistent role in the models aimed at distinguishing the three groups, suggesting their usefulness as markers of geographical origin. Many of the most important variables in the model are REEs, and their potential use as food tracers has already been determined [36,37,40]. It is worth noting that the top rankers of the random forest model are different with respect to the most influential variables found with the Wilcoxon and effect size tests. In particular, in the pairwise comparisons (Umbria versus Veneto and Umbria versus other), the isotopic parameters play a prominent role, while in the three-class classification of the random forest model, the influence of elemental and isotopic composition is much more balanced, and many elements are influential as a result. 

## 3. Materials and Methods

### 3.1. Samples and Preparation

Sixty-three samples of Virginia Bright tobacco leaves were analyzed. Of these, 20 samples were from Umbria, 10 from Veneto, and 33 from other geographical origins (Table S1). Each sample was constituted by around 30 leaves of tobacco randomly collected in a crop during 2017 and 2018. Each sample was oven-dried (70 °C) and milled (<0.5 mm). For the isotopic analysis, the samples were freeze-dried.

Before the elemental analysis, an aliquot of about 0.5 g was acid-digested after the addition of 4 mL of ultrapure, 67–69% nitric acid (Carlo Erba, Milan, Italy), by means of a microwave single-reactor chamber (Ultrawave single reactor chamber, Milestone Inc., Shelton, CT, USA) equipped with 15 mL quartz vessels, following a multi-step temperature program as described in Bertoldi et al. [41]. Mineralized solutions were transferred in PP (Poly Propylene) vials and brought to 25 mL with ultrapure water (18.2 MΩ cm) produced with a Milli-Q water producer (Millipore, Bedford, MA, USA). Each digestion batch included a blank sample (reagent only, without sample) and a certified reference material (“Peach leaves” SRM 1547, National Institute of Standards and Technology, Gaithersburg, MD, USA), in order to ensure the absence of contamination and to check for any loss of volatile elements or incomplete mineralization. All lab ware used for sample preparation for the elemental analysis were thoroughly washed with 5% nitric acid solution and doubly rinsed with ultrapure water.

### 3.2. Analytical Methods

#### 3.2.1. Stable Isotope Ratios Analysis

Around 4 mg of sample was weighted in tin capsules for the analysis of δ^13^C, δ^15^N, and δ^34^S in one run, and 0.2 mg in silver capsules for δ^18^O and δ^2^H analysis. The analyses were carried out using an Isotope Ratio Mass Spectrometer (IsoPrime, Isoprime Limited, Langenselbold, Germany) equipped with an elemental analyzer (VARIO CUBE, Isoprime Limited, Langenselbold, Germany) for the ^13^C/^12^C, ^15^N/^14^N, and ^34^S/^32^S ratio determinations. The ^2^H/^1^H and ^18^O/^16^O ratios were measured using an IRMS (Finnigan DELTA XP, Thermo Scientific, Waltham, MA, USA) coupled with a pyrolyser (Finningan DELTA TC/EA, high temperature conversion elemental analyser, Thermo Scientific, Waltham, MA, USA). The isotope ratios were expressed in δ‰ versus VPDB (Vienna Pee Dee Belemnite) for δ^13^C, VSMOW (Vienna Standard Mean Ocean Water) for δ^18^O and δ^2^H, air for δ^15^N, and VCDT (Vienna Canyon Diablo Troilite) for δ^34^S, according to the following formula:

δ*^i^E* = (*^i^R_sample_* − *^i^R_reference_*)/*^i^R_reference_*

where *R_sample_* is the isotope ratio measured for the sample, and *R_reference_* is the isotope ratio of the international standard; superscript *i* denotes the mass number of the heavier (higher atomic mass) isotope. The delta values were multiplied by 1000 and expressed in units per mil (‰). Sample analysis was carried out in duplicate. The isotopic values of δ^13^C, δ^15^N, and δ^34^S were calculated against in-house working standards (a casein and a flour for δ^13^C and δ^15^N, U.S. Geological Survey (USGS) 42 and two flours for δ^34^S), which were themselves calibrated against international reference materials: L-glutamic acid USGS 40 (U.S. Geological Survey, Reston, VA, USA), fuel oil NBS-22 (IAEA (International Atomic Energy Agency), Vienna, Austria), and sugar IAEA-CH-6 (IAEA) for ^13^C/^12^C; L-glutamic acid USGS 40 and potassium nitrate IAEA-NO3 (IAEA) for ^15^N/^14^N; and barium sulphates IAEA-SO-5 and NBS 127 (IAEA) for ^34^S/^32^S. The isotopic values of δ^2^H and δ^18^O were calculated against USGS 54 (Canadian lodgepole pine wood powder; USGS) and USGS 56 (Mexican ziricote wood powder, USGS) standards.

The precision of measurement, expressed as one standard deviation when measuring the same sample 10 times, was 0.1‰ for ^13^C/^12^C and ^15^N/^14^N, 0.3‰ for ^18^O/^16^O of bulk, 0.4‰ for ^34^S/^32^S, and 1‰ for ^2^H/^1^H.

#### 3.2.2. Elemental Analysis

The analysis of 56 elements (Li, Be, B, Na, Mg, Al, P, K, Ca, V, Cr, Mn, Fe, Co, Ni, Cu, Zn, Ga, Ge, As, Se, Rb, Sr, Y, Mo, Pd, Ag, Cd, In, Sn, Sb, Te, Cs, Ba, La, Ce, Pr, Nd, Sm, Eu, Gd, Dy, Ho, Er, Tm, Yb, Re, Ir, Pt, Au, Hg, Tl, Pb, Bi, Th, and U) was carried out with an inductively coupled plasma–mass spectrometer (ICP-MS), the Agilent 7500ce (Agilent Technologies, Santa Clara, CA, USA) equipped with quartz torch, micromist nebulizer, Ni cones, and an octopole reaction system (collision cell with He gas and reaction cell with H_2_). To minimize polyatomic interference, a collision cell was used for Na, Mg, V, Cr, Fe, Ni, Cu, Zn, As, and Eu analysis, while the reaction cell was used for Ca, Ga, and Se analysis.

Inductively coupled plasma, multi-element standard solution VI-, Ba-, Cu-, Mg-, Mn-, Na-, Ni-, and Sr-certified standard solutions were from Merck (Darmstadt, Germany). Al-, Ca-, Cs-, K-, Fe-, P-, and Rb-certified standard solutions were purchased from CPI International (Santa Rosa, CA, USA). ICP-MS calibration standard 4 solution and Sc-, Rh-, and Tb-certified standard solutions were provided by Aristar BDH (Poole, UK). Multi-element calibration standard 1 and 3 solutions, Hg standard 2A solution, and tuning solution (Li, Ce, Y, and Tl) were from Agilent Technologies (Santa Clara, CA, USA). A solution of Sc, Rh, and Tb was used as added online internal standard to correct eventual instrumental signal drift during time.

Before analysis, the sensitivity, resolution, and P/A (Pulse/Analog) factor were optimized, whereas the oxide ratio (CeO/Ce %) and double-charged ratio (Ce^++^/Ce^+^ %) were minimized, following the manufacturer’s specification and using an Li, Y, Ce, and Tl solution.

For method validation, the detection limit, repeatability, and accuracy were determined as follows. The detection limit (DL) for each element was calculated as three times the standard deviation of the signal obtained for 10 blanks analyzed in a sequence. All data were higher than the calculated DL, except for Ir, Pt, and Au, which were always below 1.5 µg/kg, and therefore are not further reported or discussed. Repeatability was determined by preparing and analyzing five samples in duplicate, and by calculating the average coefficient of variation with results below 10% for all quantified elements. Accuracy was obtained by analyzing a certified reference material in each batch, and by calculating the average recovery for each element. Recoveries ranged between 79% and 112%, and were considered satisfactory for the scope of this research.

### 3.3. Statistical Analysis

Samples’ values were analyzed with R (R Development Core Team, 2014), relying on the following packages for data handling, visualization, and analysis: tidyverse [42], FactoMineR [43], factoextra [44], reticulate [45], effsize [46]. Machine learning analysis was performed in Python by using ScikitLearn [47]. Samples were grouped in three categories on the basis of their geographical origin: Umbria, Veneto, and other. Univariate analysis was applied to compare the different geographical origins, relying on a Wilcoxon non-parametric test. The strength of the difference of the variables with statistically significant results was estimated by calculating the Cohen’s *d* effect size [48]. Two multivariate statistical approaches were applied: one unsupervised approach (principal component analysis) and a supervised classification method (random forest) [49]. PCA was primarily used to highlight the sample characteristics responsible for the larger part of the dataset variance. PCA analysis was performed on mean-centered and scaled dataset. Random forest (RF), instead, was applied to assess if the combination of isotopic and compositional data could be used to classify the samples according to their origin. RF was also used to identify the most discriminating analytical variables in a three-class classification problem. RF prediction accuracy was validated in a set of 500 training/test splits of the original dataset (0.7/0.3). As a further validation, the robustness of the results was compared with a “random region” validation scheme, in which the different samples were randomly assigned to Umbria, Veneto, and other groups. The quality of the classification was assessed by using the mcc package [50] and class-specific accuracy.

The variable importance in the model was assessed by comparing the ranks of the variable importance in the 500 training/test splits.

## 4. Conclusions

In this study, the isotopic and elemental profiles (δ^2^H, δ^13^C, δ^15^N, δ^18^O, and δ^34^S, Ag, Al, As, B, Ba, Be, Bi, Ca, Cd, Ce, Co, Cr, Cs, Cu, Dy, Er, Eu, Fe, Ga, Gd, Ge, Hg, Ho, Ir, K, La, Li, Mg, Mn, Mo, Na, Nd, Ni, P, Pb, Pd, Pr, Rb, Re, Sb, Se, Sm, Sn, Sr, Th, Tl, Tm, U, V, Y, Yb, and Zn) of Virginia Bright tobacco samples collected in Umbria, Veneto, and in other geographical areas were investigated. It was confirmed that the main factor influencing the isotopic and elemental signatures of tobacco is its geographical origin. In particular, Umbrian tobacco is characterized by an isotopic and elemental profile that makes it different from tobacco of different origins. Umbrian tobacco showed significantly higher values of δ^2^H, δ^13^C, and δ^18^O, and lower values of δ^34^S, Ag, Al, As, Be, Bi, Ce, Co, Cr, Cs, Dy, Er, Eu, Fe, Ga, Gd, Ge, Ho, In, La, Mg, Mn, Nd, Pb, Pr, Rb, Sm, Sn, Th, Tl, Tm, U, V, Y, and Yb. It is worth noting that Umbrian tobacco has a significantly lower content in elements that are potentially dangerous for human health, like As, Pb, Sn, Be, Cr, and Tl. The proposed machine learning approach has shown strong potential for the geographical classification of tobacco from Umbria on the basis of its isotopic and elemental fingerprint.

The study, if enhanced with a greater number of samples that are representative of the production, will make it possible to develop an analytical model of traceability of tobacco, which can be used to verify the geographical origin of this product on the market.

## Data Availability

Restrictions apply to the availability of these data. Data was obtained from Fattoria Autonoma Tabacchi Soc. Coop Agricola and are available from the authors with the permission of Fattoria Autonoma Tabacchi Soc. Coop Agricola.

## Supplementary Materials

The following are available online at https://www.mdpi.com/2218-1989/11/3/186/s1, Figure S1: Distribution of isotopic values (expressed in δ ‰) and elemental composition (expressed in g or mg or µg/kg, depending on the element), Table S1: Isotopic and elemental data (median, minimum and maximum values, 25th and 75th percentiles) for tobacco samples from different areas.
