# Supplementary material for: Elemental and Isotopic Characterization of Tobacco from Umbria
Source: Metabolites. 2021 Mar 22;11(3):186. doi: 10.3390/metabo11030186 (PMC8004217; doi:10.3390/metabo11030186)

**Table S1.** Isotopic and elemental data (median, minimum and maximum values, 25th and 75th percentiles) for tobacco samples from different areas.

| | Italy - Umbria N=20 | | | | |
|---|---|---|---|---|---|
| Parameter | Median | Minimum | Maximum | 25th | 75th |
| $\delta^{2}H$ ‰ vs.V-SMOW | -89 | -102 | -78 | -94 | -83 |
| $\delta^{13}C$ ‰ vs.V-PDB | -26.5 | -28.3 | -25.1 | -27.0 | -25.9 |
| $\delta^{15}N$ ‰ vs.AIR | 2.0 | -0.4 | 5.4 | 1.0 | 3.5 |
| $\delta^{18}O$ ‰ vs.V-SMOW | 27.3 | 25.3 | 29.7 | 26.8 | 27.9 |
| $\delta^{34}S$ ‰ vs.V-CDT | 1.1 | -5.9 | 5.4 | 0.0 | 3.4 |
| Li mg/kg | 12.9 | 4.3 | 23.1 | 8.1 | 18.2 |
| Be μg/kg | 3.5 | 1.8 | 19.5 | 2.6 | 7.2 |
| B mg/kg | 44.5 | 12.9 | 63.0 | 38.3 | 51.1 |
| Na mg/kg | 159 | 70 | 400 | 128 | 202 |
| Mg g/kg | 2.2 | 1.5 | 4.5 | 1.8 | 2.9 |
| Al mg/kg | 104 | 58 | 580 | 84 | 198 |
| P g/kg | 1.8 | 1.4 | 2.5 | 1.7 | 2.0 |
| K g/kg | 17.2 | 13.2 | 20.7 | 15.2 | 19.0 |
| Ca g/kg | 25.1 | 17.6 | 47.5 | 19.7 | 31.3 |
| V mg/kg | 0.18 | 0.10 | 1.22 | 0.14 | 0.37 |
| Cr mg/kg | 0.46 | 0.24 | 2.00 | 0.32 | 0.84 |
| Mn mg/kg | 37.4 | 24.7 | 88.3 | 31.7 | 43.6 |
| Fe mg/kg | 92 | 58 | 447 | 79 | 163 |
| Co mg/kg | 0.13 | 0.11 | 0.34 | 0.11 | 0.21 |
| Ni mg/kg | 0.84 | 0.59 | 1.71 | 0.62 | 1.29 |
| Cu mg/kg | 10.0 | 3.5 | 18.4 | 6.4 | 12.1 |
| Zn mg/kg | 14.0 | 5.1 | 47.5 | 10.8 | 17.6 |
| Ga μg/kg | 27.1 | 16.0 | 165.4 | 22.1 | 55.9 |
| Ge μg/kg | 3.1 | 1.9 | 13.0 | 2.5 | 5.2 |
| As μg/kg | 28.5 | 15.2 | 124.8 | 20.9 | 55.9 |
| Se μg/kg | 24.1 | 11.3 | 64.9 | 19.6 | 37.5 |
| Rb mg/kg | 3.3 | 2.0 | 6.8 | 2.5 | 4.0 |
| Sr mg/kg | 80.7 | 44.9 | 189.3 | 63.6 | 96.0 |
| Y μg/kg | 63 | 33 | 331 | 43 | 121 |
| Mo mg/kg | 0.37 | 0.12 | 0.75 | 0.32 | 0.47 |
| Pd μg/kg | 6.0 | 3.1 | 12.8 | 5.5 | 9.2 |
| Ag μg/kg | 7.5 | 4.3 | 11.7 | 6.3 | 10.2 |
| Cd mg/kg | 0.30 | 0.15 | 0.65 | 0.27 | 0.47 |
| In μg/kg | 0.43 | 0.10 | 1.18 | 0.21 | 0.62 |
| Sn μg/kg | 43.3 | 20.1 | 146.3 | 34.1 | 66.4 |
| Sb μg/kg | 370 | 160 | 794 | 323 | 564 |
| Te μg/kg | 0.62 | 0.17 | 1.73 | 0.43 | 0.91 |
| Cs μg/kg | 20.4 | 10.3 | 90.8 | 16.0 | 35.9 |
| Ba mg/kg | 30.1 | 10.5 | 55.4 | 24.8 | 41.5 |
| La μg/kg | 97 | 51 | 455 | 73 | 218 |
| Ce μg/kg | 177 | 88 | 944 | 127 | 352 |
| Pr μg/kg | 22.3 | 11.4 | 111.3 | 16.3 | 45.6 |
| Nd μg/kg | 83 | 42 | 437 | 60 | 168 |
| Sm μg/kg | 16.6 | 8.6 | 92.0 | 11.9 | 32.3 |
| Eu μg/kg | 5.4 | 2.8 | 19.6 | 4.6 | 9.3 |
| Gd μg/kg | 15.7 | 8.3 | 87.6 | 12.0 | 31.1 |
| Dy μg/kg | 10.7 | 5.4 | 62.2 | 8.0 | 19.4 |
| Ho μg/kg | 2.0 | 1.0 | 11.0 | 1.4 | 3.4 |
| Er μg/kg | 5.0 | 2.8 | 30.3 | 4.0 | 9.1 |
| Tm μg/kg | 0.54 | 0.27 | 3.06 | 0.38 | 0.93 |
| Yb μg/kg | 4.2 | 2.2 | 23.1 | 3.1 | 6.8 |
| Re μg/kg | 2.2 | 0.8 | 4.7 | 1.4 | 3.6 |
| Hg μg/kg | 27.2 | 19.2 | 130.7 | 20.9 | 34.3 |
| Tl μg/kg | 3.7 | 2.3 | 10.2 | 2.9 | 5.7 |
| Pb μg/kg | 130 | 73 | 310 | 92 | 173 |
| Bi μg/kg | 1.8 | 0.9 | 5.2 | 1.3 | 3.0 |
| Th μg/kg | 30.0 | 14.4 | 129.6 | 24.8 | 54.9 |
| U μg/kg | 6.4 | 2.7 | 17.2 | 4.7 | 8.2 |

| | Italy - Veneto N= 10 | | | | |
|---|---|---|---|---|---|
| Parameter | Median | Minimum | Maximum | 25th | 75th |
| $\delta^{2}H$ ‰ vs.V-SMOW | -108 | -116 | -98 | -112 | -103 |
| $\delta^{13}C$ ‰ vs.V-PDB | -28.3 | -29.2 | -25.8 | -28.6 | -27.2 |
| $\delta^{15}N$ ‰ vs.AIR | 1.3 | 0.7 | 4.5 | 0.8 | 3.5 |
| $\delta^{18}O$ ‰ vs.V-SMOW | 24.3 | 22.7 | 27.1 | 23.2 | 25.3 |
| $\delta^{34}S$ ‰ vs.V-CDT | 6.4 | 3.3 | 8.6 | 4.9 | 7.3 |
| Li mg/kg | 2.4 | 1.1 | 11.8 | 1.2 | 5.4 |
| Be μg/kg | 6.4 | 2.3 | 19.5 | 4.0 | 9.0 |
| B mg/kg | 34.1 | 15.3 | 48.0 | 28.3 | 46.0 |
| Na mg/kg | 85 | 48 | 129 | 68 | 114 |
| Mg g/kg | 3.4 | 1.4 | 5.1 | 2.6 | 4.0 |
| Al mg/kg | 217 | 96 | 587 | 141 | 312 |
| P g/kg | 1.7 | 1.1 | 2.1 | 1.3 | 2.0 |
| K g/kg | 20.8 | 15.5 | 28.0 | 18.4 | 24.0 |
| Ca g/kg | 31.9 | 19.7 | 44.2 | 25.0 | 38.3 |
| V mg/kg | 0.36 | 0.17 | 1.14 | 0.25 | 0.66 |
| Cr mg/kg | 0.97 | 0.70 | 3.17 | 0.77 | 1.74 |
| Mn mg/kg | 23.1 | 12.9 | 73.2 | 16.8 | 33.2 |
| Fe mg/kg | 165 | 86 | 529 | 124 | 263 |
| Co mg/kg | 0.13 | 0.08 | 0.25 | 0.10 | 0.25 |
| Ni mg/kg | 0.56 | 0.40 | 1.33 | 0.43 | 1.11 |
| Cu mg/kg | 3.8 | 1.6 | 11.6 | 3.2 | 8.1 |
| Zn mg/kg | 5.3 | 3.6 | 16.3 | 3.8 | 10.0 |
| Ga μg/kg | 58.4 | 27.0 | 199.5 | 38.0 | 86.1 |
| Ge μg/kg | 5.2 | 3.1 | 15.1 | 3.8 | 8.5 |
| As μg/kg | 146.5 | 69.5 | 327.2 | 112.4 | 262.0 |
| Se μg/kg | 26.4 | 13.2 | 46.4 | 17.4 | 32.6 |
| Rb mg/kg | 1.5 | 0.7 | 4.5 | 1.0 | 2.6 |
| Sr mg/kg | 51.8 | 23.6 | 110.9 | 37.2 | 80.2 |
| Y μg/kg | 140 | 61 | 583 | 83 | 292 |
| Mo mg/kg | 1.22 | 0.83 | 2.38 | 1.05 | 1.79 |
| Pd μg/kg | 10.7 | 7.0 | 29.5 | 8.1 | 16.6 |
| Ag μg/kg | 10.0 | 3.5 | 19.2 | 4.4 | 16.6 |
| Cd mg/kg | 0.52 | 0.35 | 1.52 | 0.39 | 0.87 |
| In μg/kg | 0.86 | 0.40 | 1.78 | 0.50 | 1.02 |
| Sn μg/kg | 100.8 | 68.8 | 193.6 | 78.2 | 133.0 |
| Sb μg/kg | 272 | 155 | 511 | 242 | 310 |
| Te μg/kg | 0.87 | 0.49 | 1.52 | 0.67 | 1.20 |
| Cs μg/kg | 33.4 | 14.5 | 129.2 | 23.1 | 67.0 |
| Ba mg/kg | 28.5 | 13.5 | 96.3 | 21.6 | 42.2 |
| La μg/kg | 225 | 130 | 954 | 132 | 483 |
| Ce μg/kg | 417 | 227 | 1814 | 241 | 971 |
| Pr μg/kg | 52.1 | 28.0 | 210.7 | 30.7 | 108.1 |
| Nd μg/kg | 191 | 101 | 823 | 113 | 407 |
| Sm μg/kg | 37.6 | 19.7 | 160.3 | 22.5 | 79.0 |
| Eu μg/kg | 9.3 | 4.4 | 28.5 | 6.1 | 15.6 |
| Gd μg/kg | 36.2 | 18.6 | 149.0 | 20.7 | 74.5 |
| Dy μg/kg | 25.6 | 10.4 | 106.8 | 14.5 | 49.0 |
| Ho μg/kg | 4.5 | 1.9 | 18.2 | 2.5 | 8.7 |
| Er μg/kg | 12.1 | 5.5 | 52.6 | 6.6 | 26.4 |
| Tm μg/kg | 1.23 | 0.53 | 5.42 | 0.69 | 2.87 |
| Yb μg/kg | 9.0 | 4.3 | 43.3 | 5.4 | 25.3 |
| Re μg/kg | 2.6 | 1.5 | 4.9 | 1.8 | 3.4 |
| Hg μg/kg | 43.1 | 20.5 | 64.8 | 30.6 | 50.0 |
| Tl μg/kg | 6.1 | 3.1 | 10.5 | 4.6 | 7.2 |
| Pb μg/kg | 293 | 193 | 839 | 224 | 579 |
| Bi μg/kg | 5.7 | 3.8 | 8.5 | 4.6 | 6.6 |
| Th μg/kg | 65.8 | 31.3 | 256.1 | 39.1 | 106.5 |
| U μg/kg | 13.4 | 6.3 | 35.6 | 9.1 | 19.8 |

| | Spain N=4 | | | | |
|---|---|---|---|---|---|
| Parameter | Median | Minimum | Maximum | 25th | 75th |
| $\delta^{2}H$ ‰ vs.V-SMOW | -93 | -97 | -88 | -97 | -89 |
| $\delta^{13}C$ ‰ vs.V-PDB | -26.6 | -27.4 | -25.2 | -27.2 | -25.7 |
| $\delta^{15}N$ ‰ vs.AIR | 4.0 | 2.8 | 6.5 | 3.1 | 5.5 |
| $\delta^{18}O$ ‰ vs.V-SMOW | 30.2 | 26.9 | 34.5 | 28.3 | 32.6 |
| $\delta^{34}S$ ‰ vs.V-CDT | 6.0 | 3.9 | 9.0 | 4.7 | 7.8 |
| Li mg/kg | 7.6 | 5.3 | 12.7 | 5.6 | 11.0 |
| Be μg/kg | 24.3 | 19.7 | 28.1 | 21.1 | 27.1 |
| B mg/kg | 61.6 | 52.6 | 70.8 | 56.9 | 66.4 |
| Na mg/kg | 308 | 139 | 493 | 205 | 418 |
| Mg g/kg | 4.4 | 2.4 | 6.4 | 3.1 | 5.7 |
| Al mg/kg | 381 | 236 | 400 | 305 | 394 |
| P g/kg | 2.8 | 2.0 | 3.1 | 2.2 | 3.0 |
| K g/kg | 26.4 | 15.4 | 36.0 | 20.7 | 31.4 |
| Ca g/kg | 22.6 | 11.9 | 26.1 | 16.1 | 25.5 |
| V mg/kg | 0.38 | 0.21 | 0.43 | 0.29 | 0.41 |
| Cr mg/kg | 0.74 | 0.29 | 0.92 | 0.45 | 0.89 |
| Mn mg/kg | 90.4 | 52.8 | 196.3 | 65.3 | 149.6 |
| Fe mg/kg | 217 | 129 | 228 | 172 | 223 |
| Co mg/kg | 0.14 | 0.13 | 0.16 | 0.13 | 0.16 |
| Ni mg/kg | 0.41 | 0.24 | 0.48 | 0.32 | 0.45 |
| Cu mg/kg | 3.1 | 1.5 | 5.7 | 2.1 | 4.6 |
| Zn mg/kg | 16.5 | 6.8 | 22.7 | 11.2 | 20.1 |
| Ga μg/kg | 117.9 | 76.0 | 140.2 | 96.9 | 129.1 |
| Ge μg/kg | 8.0 | 5.1 | 8.3 | 6.5 | 8.2 |
| As μg/kg | 334.6 | 177.7 | 531.0 | 215.2 | 473.8 |
| Se μg/kg | 13.7 | 8.1 | 14.8 | 10.5 | 14.6 |
| Rb mg/kg | 7.2 | 4.2 | 8.6 | 5.4 | 8.2 |
| Sr mg/kg | 77.3 | 74.7 | 99.0 | 75.6 | 88.6 |
| Y μg/kg | 254 | 197 | 287 | 222 | 274 |
| Mo mg/kg | 0.87 | 0.32 | 1.06 | 0.52 | 1.04 |
| Pd μg/kg | 6.0 | 4.4 | 8.2 | 4.9 | 7.3 |
| Ag μg/kg | 14.9 | 7.4 | 19.6 | 9.0 | 19.4 |
| Cd mg/kg | 0.25 | 0.20 | 0.41 | 0.22 | 0.34 |
| In μg/kg | 0.98 | 0.45 | 1.41 | 0.67 | 1.24 |
| Sn μg/kg | 86.7 | 53.6 | 130.1 | 68.7 | 109.8 |
| Sb μg/kg | 238 | 219 | 297 | 224 | 272 |
| Te μg/kg | 0.91 | 0.43 | 1.15 | 0.64 | 1.06 |
| Cs μg/kg | 84.9 | 56.3 | 108.9 | 70.1 | 97.4 |
| Ba mg/kg | 18.4 | 15.6 | 36.5 | 16.8 | 27.6 |
| La μg/kg | 628 | 567 | 812 | 570 | 747 |
| Ce μg/kg | 1181 | 1024 | 1663 | 1088 | 1437 |
| Pr μg/kg | 135.0 | 125.7 | 200.0 | 125.9 | 172.0 |
| Nd μg/kg | 522 | 467 | 772 | 478 | 664 |
| Sm μg/kg | 101.5 | 81.1 | 148.4 | 88.1 | 128.1 |
| Eu μg/kg | 12.3 | 9.5 | 14.2 | 10.4 | 13.8 |
| Gd μg/kg | 85.1 | 74.0 | 117.7 | 79.0 | 101.9 |
| Dy μg/kg | 44.4 | 36.6 | 59.6 | 40.0 | 52.5 |
| Ho μg/kg | 7.1 | 5.9 | 8.6 | 6.4 | 8.0 |
| Er μg/kg | 18.1 | 15.0 | 22.3 | 16.0 | 20.7 |
| Tm μg/kg | 1.65 | 1.32 | 1.99 | 1.46 | 1.85 |
| Yb μg/kg | 11.7 | 8.7 | 14.1 | 9.9 | 13.2 |
| Re μg/kg | 1.5 | 1.2 | 4.0 | 1.2 | 2.8 |
| Hg μg/kg | 30.8 | 15.2 | 34.7 | 21.6 | 34.1 |
| Tl μg/kg | 18.0 | 10.7 | 31.6 | 12.9 | 26.3 |
| Pb μg/kg | 386 | 214 | 485 | 276 | 460 |
| Bi μg/kg | 5.2 | 2.8 | 5.5 | 3.9 | 5.5 |
| Th μg/kg | 236.1 | 127.2 | 425.0 | 154.6 | 357.7 |
| U μg/kg | 40.0 | 28.9 | 62.5 | 29.9 | 55.8 |

| | Greece N=7 | | | | |
|---|---|---|---|---|---|
| Parameter | Median | Minimum | Maximum | 25th | 75th |
| $\delta^{2}H$ ‰ vs.V-SMOW | -104 | -115 | -53 | -112 | -96 |
| $\delta^{13}C$ ‰ vs.V-PDB | -27.2 | -28.2 | -26.0 | -27.9 | -26.7 |
| $\delta^{15}N$ ‰ vs.AIR | 3.9 | 0.8 | 6.6 | 1.9 | 4.9 |
| $\delta^{18}O$ ‰ vs.V-SMOW | 27.3 | 23.9 | 32.8 | 26.2 | 29.1 |
| $\delta^{34}S$ ‰ vs.V-CDT | 5.1 | 0.9 | 6.6 | 1.9 | 6.5 |
| Li mg/kg | 15.8 | 5.7 | 20.0 | 7.1 | 19.6 |
| Be μg/kg | 8.1 | 4.0 | 37.2 | 5.4 | 10.1 |
| B mg/kg | 35.2 | 21.3 | 50.2 | 23.2 | 37.7 |
| Na mg/kg | 70 | 43 | 465 | 48 | 114 |
| Mg g/kg | 4.6 | 2.7 | 7.1 | 4.1 | 7.1 |
| Al mg/kg | 321 | 130 | 1482 | 210 | 382 |
| P g/kg | 1.9 | 1.2 | 2.1 | 1.3 | 2.0 |
| K g/kg | 14.6 | 6.2 | 16.9 | 9.4 | 16.5 |
| Ca g/kg | 24.5 | 13.7 | 31.6 | 15.9 | 27.9 |
| V mg/kg | 0.70 | 0.27 | 4.82 | 0.48 | 0.97 |
| Cr mg/kg | 2.88 | 0.96 | 8.45 | 1.85 | 8.00 |
| Mn mg/kg | 37.8 | 24.0 | 65.1 | 24.0 | 54.1 |
| Fe mg/kg | 299 | 116 | 1150 | 206 | 381 |
| Co mg/kg | 0.34 | 0.21 | 0.85 | 0.31 | 0.46 |
| Ni mg/kg | 5.09 | 2.38 | 7.42 | 3.65 | 6.53 |
| Cu mg/kg | 5.7 | 3.1 | 11.0 | 4.8 | 10.2 |
| Zn mg/kg | 16.2 | 4.8 | 21.5 | 10.6 | 19.0 |
| Ga μg/kg | 89.0 | 35.5 | 455.9 | 55.2 | 119.1 |
| Ge μg/kg | 8.4 | 3.4 | 31.7 | 5.9 | 9.3 |
| As μg/kg | 56.7 | 30.4 | 188.6 | 49.5 | 108.3 |
| Se μg/kg | 32.5 | 13.9 | 166.3 | 15.3 | 45.5 |
| Rb mg/kg | 11.8 | 4.0 | 21.7 | 8.9 | 15.5 |
| Sr mg/kg | 56.8 | 46.1 | 138.3 | 50.3 | 60.5 |
| Y μg/kg | 223 | 92 | 1021 | 159 | 370 |
| Mo mg/kg | 0.44 | 0.20 | 1.63 | 0.27 | 0.77 |
| Pd μg/kg | 13.1 | 8.9 | 18.7 | 9.2 | 14.6 |
| Ag μg/kg | 12.1 | 6.9 | 16.6 | 8.8 | 15.8 |
| Cd mg/kg | 0.63 | 0.45 | 0.94 | 0.46 | 0.70 |
| In μg/kg | 0.63 | 0.37 | 2.79 | 0.48 | 1.70 |
| Sn μg/kg | 85.7 | 32.6 | 271.2 | 35.9 | 107.4 |
| Sb μg/kg | 362 | 69 | 463 | 246 | 401 |
| Te μg/kg | 0.83 | 0.61 | 1.74 | 0.63 | 1.13 |
| Cs μg/kg | 37.8 | 16.6 | 326.0 | 32.6 | 151.5 |
| Ba mg/kg | 20.5 | 11.8 | 29.6 | 15.3 | 28.0 |
| La μg/kg | 293 | 173 | 1391 | 192 | 460 |
| Ce μg/kg | 536 | 224 | 2653 | 318 | 925 |
| Pr μg/kg | 66.6 | 32.8 | 311.3 | 43.7 | 103.1 |
| Nd μg/kg | 256 | 119 | 1218 | 166 | 393 |
| Sm μg/kg | 50.6 | 21.9 | 246.5 | 33.6 | 76.7 |
| Eu μg/kg | 13.1 | 5.9 | 57.7 | 9.4 | 17.4 |
| Gd μg/kg | 50.0 | 22.1 | 236.6 | 33.6 | 78.6 |
| Dy μg/kg | 35.8 | 13.7 | 176.7 | 25.4 | 55.4 |
| Ho μg/kg | 6.3 | 2.5 | 31.9 | 4.6 | 10.4 |
| Er μg/kg | 18.2 | 6.5 | 94.0 | 12.5 | 31.5 |
| Tm μg/kg | 1.80 | 0.69 | 6.11 | 1.31 | 3.23 |
| Yb μg/kg | 14.2 | 5.1 | 77.9 | 9.6 | 27.6 |
| Re μg/kg | 0.7 | 0.2 | 4.0 | 0.3 | 2.3 |
| Hg μg/kg | 16.5 | 14.4 | 23.2 | 14.5 | 17.8 |
| Tl μg/kg | 12.1 | 6.7 | 30.4 | 9.7 | 18.3 |
| Pb μg/kg | 185 | 121 | 628 | 127 | 235 |
| Bi μg/kg | 1.6 | 1.0 | 6.4 | 1.2 | 3.5 |
| Th μg/kg | 77.1 | 28.9 | 323.0 | 48.9 | 119.9 |
| U μg/kg | 11.6 | 3.8 | 64.2 | 7.7 | 21.0 |

| | Bulgaria N=4 | | | | |
|---|---|---|---|---|---|
| Parameter | Median | Minimum | Maximum | 25th | 75th |
| $\delta^{2}H$ ‰ vs.V-SMOW | -106 | -119 | -105 | -113 | -105 |
| $\delta^{13}C$ ‰ vs.V-PDB | -26.7 | -27.2 | -26.3 | -27.1 | -26.4 |
| $\delta^{15}N$ ‰ vs.AIR | 3.8 | 2.9 | 6.9 | 3.1 | 5.7 |
| $\delta^{18}O$ ‰ vs.V-SMOW | 24.7 | 22.4 | 25.8 | 23.1 | 25.7 |
| $\delta^{34}S$ ‰ vs.V-CDT | 4.6 | 2.9 | 5.5 | 3.7 | 5.1 |
| Li mg/kg | 20.7 | 8.3 | 98.4 | 11.9 | 62.1 |
| Be μg/kg | 11.8 | 5.6 | 18.8 | 5.8 | 18.1 |
| B mg/kg | 56.8 | 32.6 | 66.9 | 40.6 | 66.0 |
| Na mg/kg | 80 | 50 | 160 | 63 | 122 |
| Mg g/kg | 5.8 | 3.3 | 6.8 | 4.5 | 6.4 |
| Al mg/kg | 387 | 226 | 501 | 254 | 497 |
| P g/kg | 1.5 | 1.3 | 1.6 | 1.4 | 1.5 |
| K g/kg | 10.3 | 4.5 | 16.5 | 7.1 | 13.7 |
| Ca g/kg | 32.9 | 26.2 | 55.3 | 27.4 | 46.2 |
| V mg/kg | 0.94 | 0.53 | 1.17 | 0.68 | 1.11 |
| Cr mg/kg | 1.42 | 1.23 | 1.95 | 1.31 | 1.70 |
| Mn mg/kg | 71.5 | 55.6 | 73.7 | 62.7 | 73.4 |
| Fe mg/kg | 319 | 217 | 430 | 246 | 397 |
| Co mg/kg | 0.30 | 0.25 | 0.33 | 0.26 | 0.32 |
| Ni mg/kg | 2.29 | 0.91 | 4.29 | 0.92 | 3.97 |
| Cu mg/kg | 13.1 | 10.4 | 16.0 | 11.4 | 14.9 |
| Zn mg/kg | 16.1 | 13.9 | 23.6 | 14.6 | 20.3 |
| Ga μg/kg | 107.3 | 75.9 | 147.1 | 79.1 | 139.7 |
| Ge μg/kg | 10.3 | 7.3 | 12.0 | 8.1 | 11.9 |
| As μg/kg | 106.4 | 82.2 | 144.3 | 85.3 | 134.3 |
| Se μg/kg | 72.1 | 21.6 | 157.8 | 46.5 | 115.3 |
| Rb mg/kg | 7.9 | 5.4 | 21.6 | 6.5 | 14.9 |
| Sr mg/kg | 154.0 | 97.2 | 230.3 | 116.5 | 201.2 |
| Y μg/kg | 266 | 142 | 372 | 153 | 370 |
| Mo mg/kg | 0.51 | 0.09 | 0.79 | 0.23 | 0.72 |
| Pd μg/kg | 15.9 | 7.8 | 26.0 | 9.1 | 23.7 |
| Ag μg/kg | 32.3 | 18.6 | 40.7 | 22.5 | 39.4 |
| Cd mg/kg | 0.76 | 0.31 | 1.24 | 0.40 | 1.14 |
| In μg/kg | 0.96 | 0.69 | 2.62 | 0.81 | 1.80 |
| Sn μg/kg | 85.1 | 53.0 | 162.5 | 66.5 | 126.4 |
| Sb μg/kg | 686 | 213 | 996 | 336 | 954 |
| Te μg/kg | 1.93 | 1.50 | 3.04 | 1.54 | 2.66 |
| Cs μg/kg | 69.4 | 49.2 | 79.0 | 59.3 | 74.3 |
| Ba mg/kg | 48.9 | 32.1 | 53.1 | 38.8 | 52.7 |
| La μg/kg | 474 | 244 | 696 | 252 | 691 |
| Ce μg/kg | 765 | 442 | 1158 | 458 | 1108 |
| Pr μg/kg | 89.7 | 53.7 | 135.0 | 56.7 | 127.4 |
| Nd μg/kg | 334 | 195 | 519 | 208 | 483 |
| Sm μg/kg | 63.4 | 38.0 | 101.4 | 40.8 | 92.3 |
| Eu μg/kg | 16.9 | 12.1 | 25.4 | 12.7 | 23.0 |
| Gd μg/kg | 63.3 | 36.9 | 96.9 | 39.9 | 90.2 |
| Dy μg/kg | 41.5 | 22.7 | 61.7 | 25.0 | 58.7 |
| Ho μg/kg | 7.5 | 4.1 | 10.6 | 4.4 | 10.4 |
| Er μg/kg | 22.1 | 11.1 | 30.6 | 12.4 | 30.6 |
| Tm μg/kg | 2.13 | 1.11 | 3.20 | 1.23 | 3.05 |
| Yb μg/kg | 17.2 | 8.4 | 25.0 | 9.6 | 24.3 |
| Re μg/kg | 2.6 | 0.4 | 263.4 | 1.2 | 133.3 |
| Hg μg/kg | 29.7 | 27.4 | 37.6 | 28.0 | 34.2 |
| Tl μg/kg | 12.2 | 7.0 | 25.2 | 9.3 | 19.1 |
| Pb μg/kg | 866 | 324 | 1368 | 474 | 1238 |
| Bi μg/kg | 4.4 | 3.1 | 9.6 | 3.4 | 7.3 |
| Th μg/kg | 97.9 | 62.2 | 136.2 | 64.7 | 132.5 |
| U μg/kg | 13.5 | 7.9 | 23.0 | 8.6 | 20.4 |

Table S1 continued.

| | Hungary N=3 | | | | | Poland N=3 | | | | | Africa N=3 | | | | | Argentina N=1 | Brazil N=1 | USA N=1 | India N=2 | China N=2 | Asia N=1 |
|---|---|---|---|---|---|---|---|---|---|---|---|---|---|---|---|---|---|---|---|---|---|
| Parameter | Median | Minimum | Maximum | 25th | 75th | Median | Minimum | Maximum | 25th | 75th | Median | Minimum | Maximum | 25th | 75th | | | | Median | Median | |
| $\delta^{2}H$ ‰ vs. V-SMOW | -111 | -111 | -108 | -111 | -108 | -112 | -136 | -92 | -136 | -92 | -104 | -106 | -71 | -106 | -71 | -95 | -68 | -99 | -86 | -114 | -85 |
| $\delta^{13}C$ ‰ vs. V-PDB | -27.5 | -27.6 | -26.4 | -27.6 | -26.4 | -27.5 | -28.0 | -25.6 | -28.0 | -25.6 | -27.9 | -28.2 | -27.0 | -28.2 | -27.0 | -26.8 | -27.6 | -27.6 | -26.7 | -27.8 | -27.7 |
| $\delta^{15}N$ ‰ vs. AIR | 1.9 | 1.0 | 2.2 | 1.0 | 2.2 | 4.5 | 2.2 | 9.3 | 2.2 | 9.3 | 0.2 | -1.2 | 0.7 | -1.2 | 0.7 | 2.6 | 1.0 | 1.1 | 2.4 | -0.2 | 0.8 |
| $\delta^{18}O$ ‰ vs. V-SMOW | 24.6 | 24.4 | 25.3 | 24.4 | 25.3 | 20.3 | 19.6 | 23.6 | 19.6 | 23.6 | 20.4 | 20.1 | 25.6 | 20.1 | 25.6 | 22.2 | 22.9 | 23.0 | 24.8 | 16.8 | 23.7 |
| $\delta^{34}S$ ‰ vs. V-CDT | 4.1 | 3.7 | 4.7 | 3.7 | 4.7 | 2.7 | 2.1 | 3.0 | 2.1 | 3.0 | 3.4 | 3.3 | 8.0 | 3.3 | 8.0 | 6.3 | 6.3 | 9.8 | 6.6 | 5.2 | 4.6 |
| Li mg/kg | 15.1 | 8.5 | 68.9 | 8.5 | 68.9 | 0.8 | 0.7 | 1.5 | 0.7 | 1.5 | 6.3 | 2.9 | 11.1 | 2.9 | 11.1 | 107.6 | 5.6 | 2.2 | 15.8 | 6.8 | 41.1 |
| Be ug/kg | 21.5 | 9.2 | 24.3 | 9.2 | 24.3 | 19.2 | 15.1 | 32.3 | 15.1 | 32.3 | 34.5 | 20.9 | 43.1 | 20.9 | 43.1 | 21.2 | 30.5 | 9.8 | 15.2 | 35.0 | 21.6 |
| B mg/kg | 47.5 | 40.8 | 51.4 | 40.8 | 51.4 | 32.6 | 17.2 | 37.8 | 17.2 | 37.8 | 23.5 | 22.4 | 27.2 | 22.4 | 27.2 | 65.3 | 15.9 | 35.4 | 51.2 | 22.2 | 22.5 |
| Na mg/kg | 24 | 16 | 28 | 16 | 28 | 36 | 34 | 48 | 34 | 48 | 39 | 16 | 43 | 16 | 43 | 92.2 | 117.8 | 69.4 | 183.1 | 37.2 | 367.9 |
| Mg g/kg | 1.8 | 1.5 | 1.8 | 1.5 | 1.8 | 2.9 | 2.2 | 4.2 | 2.2 | 4.2 | 4.1 | 3.6 | 4.8 | 3.6 | 4.8 | 4.8 | 9.4 | 6.7 | 5.6 | 4.5 | 8.2 |
| Al mg/kg | 159 | 100 | 176 | 100 | 176 | 715 | 375 | 982 | 375 | 982 | 476 | 294 | 745 | 294 | 745 | 417.9 | 736.5 | 378.7 | 414.2 | 669.0 | 601.5 |
| P g/kg | 2.2 | 2.1 | 2.3 | 2.1 | 2.3 | 2.2 | 1.7 | 4.7 | 1.7 | 4.7 | 2.2 | 1.7 | 2.7 | 1.7 | 2.7 | 1.7 | 1.5 | 1.5 | 1.9 | 1.3 | 1.3 |
| K g/kg | 21.1 | 16.4 | 25.7 | 16.4 | 25.7 | 19.0 | 13.2 | 19.2 | 13.2 | 19.2 | 32.4 | 27.8 | 42.7 | 27.8 | 42.7 | 23.1 | 29.1 | 25.1 | 17.5 | 31.2 | 16.5 |
| Ca g/kg | 21.3 | 16.4 | 21.3 | 16.4 | 21.3 | 34.7 | 28.9 | 41.2 | 28.9 | 41.2 | 17.0 | 16.3 | 18.1 | 16.3 | 18.1 | 23.3 | 26.2 | 20.4 | 21.5 | 22.1 | 44.4 |
| V mg/kg | 0.26 | 0.17 | 0.30 | 0.17 | 0.30 | 1.31 | 0.79 | 1.80 | 0.79 | 1.80 | 0.48 | 0.19 | 0.94 | 0.19 | 0.94 | 0.65 | 0.92 | 0.49 | 0.90 | 1.37 | 1.34 |
| Cr mg/kg | 0.57 | 0.44 | 0.63 | 0.44 | 0.63 | 3.21 | 2.95 | 6.00 | 2.95 | 6.00 | 1.13 | 0.81 | 2.03 | 0.81 | 2.03 | 1.00 | 0.98 | 1.09 | 1.70 | 1.44 | 1.80 |
| Mn mg/kg | 592.6 | 425.5 | 830.8 | 425.5 | 830.8 | 66.8 | 47.8 | 171.3 | 47.8 | 171.3 | 301.6 | 162.1 | 310.8 | 162.1 | 310.8 | 81.6 | 227.8 | 85.3 | 235.6 | 330.3 | 109.2 |
| Fe mg/kg | 194 | 130 | 202 | 130 | 202 | 519 | 359 | 644 | 359 | 644 | 212 | 90 | 454 | 90 | 454 | 312.1 | 409.3 | 188.4 | 420.6 | 487.9 | 592.4 |
| Co mg/kg | 0.78 | 0.77 | 1.39 | 0.77 | 1.39 | 0.35 | 0.33 | 0.37 | 0.33 | 0.37 | 0.73 | 0.65 | 0.94 | 0.65 | 0.94 | 0.25 | 0.30 | 0.26 | 1.17 | 1.00 | 0.64 |
| Ni mg/kg | 4.34 | 1.68 | 6.38 | 1.68 | 6.38 | 1.46 | 0.97 | 1.75 | 0.97 | 1.75 | 1.35 | 1.29 | 1.88 | 1.29 | 1.88 | 1.12 | 0.62 | 0.69 | 3.05 | 1.40 | 1.53 |
| Cu mg/kg | 17.4 | 13.0 | 19.7 | 13.0 | 19.7 | 3.3 | 2.9 | 5.8 | 2.9 | 5.8 | 6.2 | 4.9 | 9.7 | 4.9 | 9.7 | 4.9 | 3.4 | 7.5 | 6.7 | 5.7 | 8.9 |
| Zn mg/kg | 30.9 | 24.9 | 46.4 | 24.9 | 46.4 | 12.8 | 9.4 | 56.5 | 9.4 | 56.5 | 17.4 | 9.0 | 25.3 | 9.0 | 25.3 | 5.4 | 18.0 | 26.3 | 11.3 | 36.1 | 8.1 |
| Ga ug/kg | 41.9 | 32.4 | 45.8 | 32.4 | 45.8 | 216.0 | 138.5 | 328.5 | 138.5 | 328.5 | 113.0 | 68.1 | 194.3 | 68.1 | 194.3 | 112.9 | 177.5 | 102.7 | 110.3 | 177.7 | 152.4 |
| Ge ug/kg | 5.0 | 3.7 | 5.0 | 3.7 | 5.0 | 25.0 | 22.0 | 28.3 | 22.0 | 28.3 | 10.5 | 8.4 | 17.1 | 8.4 | 17.1 | 8.5 | 11.9 | 8.1 | 9.7 | 15.8 | 13.6 |
| As ug/kg | 93.3 | 65.7 | 93.4 | 65.7 | 93.4 | 209.0 | 178.9 | 212.0 | 178.9 | 212.0 | 59.3 | 39.7 | 77.9 | 39.7 | 77.9 | 198.0 | 113.9 | 126.1 | 68.3 | 404.1 | 105.8 |
| Se ug/kg | 24.5 | 19.8 | 34.5 | 19.8 | 34.5 | 57.4 | 32.5 | 61.3 | 32.5 | 61.3 | 19.2 | 15.7 | 35.8 | 15.7 | 35.8 | 29.2 | 15.2 | 28.6 | 71.5 | 59.2 | 42.7 |
| Rb mg/kg | 4.5 | 4.2 | 5.6 | 4.2 | 5.6 | 6.3 | 5.0 | 6.5 | 5.0 | 6.5 | 42.9 | 18.7 | 45.0 | 18.7 | 45.0 | 5.3 | 26.0 | 10.5 | 9.4 | 10.2 | 14.2 |
| Sr mg/kg | 74.2 | 72.5 | 105.5 | 72.5 | 105.5 | 99.3 | 90.2 | 126.3 | 90.2 | 126.3 | 196.7 | 77.6 | 203.7 | 77.6 | 203.7 | 164.2 | 137.2 | 51.9 | 129.3 | 69.9 | 290.4 |
| Y ug/kg | 360 | 292 | 595 | 292 | 595 | 639 | 453 | 1064 | 453 | 1064 | 693 | 668 | 2081 | 668 | 2081 | 258.1 | 414.2 | 678.5 | 518.4 | 836.8 | 466.2 |
| Mo mg/kg | 0.23 | 0.14 | 0.34 | 0.14 | 0.34 | 0.99 | 0.98 | 2.14 | 0.98 | 2.14 | 0.38 | 0.36 | 0.61 | 0.36 | 0.61 | 0.87 | 0.26 | 0.37 | 0.44 | 0.32 | 0.35 |
| Pd ug/kg | 22.4 | 15.3 | 40.2 | 15.3 | 40.2 | 36.1 | 32.3 | 37.4 | 32.3 | 37.4 | 4.9 | 4.4 | 5.0 | 4.4 | 5.0 | 7.9 | 25.6 | 20.0 | 9.2 | 139.1 | 8.0 |
| Ag ug/kg | 51.1 | 21.5 | 63.4 | 21.5 | 63.4 | 13.1 | 9.2 | 20.0 | 9.2 | 20.0 | 15.3 | 11.1 | 20.4 | 11.1 | 20.4 | 7.7 | 10.3 | 8.6 | 22.3 | 17.7 | 17.8 |
| Cd mg/kg | 1.15 | 0.79 | 2.10 | 0.79 | 2.10 | 1.75 | 1.40 | 1.77 | 1.40 | 1.77 | 0.17 | 0.15 | 0.21 | 0.15 | 0.21 | 0.38 | 1.21 | 1.05 | 0.43 | 8.26 | 0.35 |
| In ug/kg | 0.41 | 0.24 | 0.68 | 0.24 | 0.68 | 1.71 | 1.39 | 2.31 | 1.39 | 2.31 | 0.41 | 0.13 | 0.59 | 0.13 | 0.59 | 0.51 | 0.86 | 0.47 | 3.78 | 4.05 | 0.92 |
| Sn ug/kg | 55.9 | 37.9 | 60.9 | 37.9 | 60.9 | 147.7 | 122.2 | 179.7 | 122.2 | 179.7 | 21.1 | 16.6 | 29.3 | 16.6 | 29.3 | 32.2 | 50.3 | 45.3 | 27.2 | 106.8 | 34.4 |
| Sb ug/kg | 424 | 280 | 444 | 280 | 444 | 497 | 327 | 1137 | 327 | 1137 | 1114 | 587 | 1375 | 587 | 1375 | 802.4 | 371.1 | 640.3 | 968.1 | 1222.2 | 601.0 |
| Te ug/kg | 1.33 | 0.94 | 1.52 | 0.94 | 1.52 | 2.21 | 1.80 | 2.61 | 1.80 | 2.61 | 1.38 | 0.85 | 2.05 | 0.85 | 2.05 | 1.32 | 1.65 | 1.11 | 1.47 | 2.38 | 1.68 |
| Cs ug/kg | 20.0 | 13.6 | 20.2 | 13.6 | 20.2 | 96.2 | 57.7 | 120.7 | 57.7 | 120.7 | 98.5 | 61.9 | 137.7 | 61.9 | 137.7 | 118.2 | 139.4 | 47.9 | 42.9 | 133.8 | 85.6 |
| Ba mg/kg | 58.8 | 38.0 | 66.2 | 38.0 | 66.2 | 37.1 | 27.8 | 43.5 | 27.8 | 43.5 | 145.5 | 121.8 | 147.5 | 121.8 | 147.5 | 69.0 | 106.6 | 49.7 | 55.5 | 44.1 | 115.4 |
| La ug/kg | 856 | 645 | 1014 | 645 | 1014 | 1362 | 1087 | 2376 | 1087 | 2376 | 3391 | 2518 | 5485 | 2518 | 5485 | 489.9 | 1515.1 | 2935.6 | 1087.0 | 2172.0 | 839.2 |
| Ce ug/kg | 965 | 626 | 1140 | 626 | 1140 | 2561 | 1634 | 4613 | 1634 | 4613 | 2687 | 2585 | 3086 | 2585 | 3086 | 1002.7 | 2343.2 | 3142.5 | 1441.0 | 2707.0 | 1626.1 |
| Pr ug/kg | 97.4 | 69.5 | 127.4 | 69.5 | 127.4 | 292.9 | 196.7 | 557.3 | 196.7 | 557.3 | 428.6 | 324.3 | 828.4 | 324.3 | 828.4 | 112.3 | 197.2 | 331.6 | 161.6 | 280.1 | 160.8 |
| Nd ug/kg | 364 | 253 | 486 | 253 | 486 | 1099 | 721 | 1953 | 721 | 1953 | 1540 | 1207 | 3066 | 1207 | 3066 | 423.6 | 718.8 | 1211.2 | 606.5 | 1083.4 | 611.6 |
| Sm ug/kg | 60.1 | 40.9 | 81.4 | 40.9 | 81.4 | 204.2 | 125.9 | 361.7 | 125.9 | 361.7 | 206.6 | 158.7 | 462.1 | 158.7 | 462.1 | 81.2 | 113.5 | 153.2 | 104.3 | 161.9 | 114.3 |
| Eu ug/kg | 17.0 | 14.5 | 22.6 | 14.5 | 22.6 | 31.7 | 24.0 | 48.8 | 24.0 | 48.8 | 50.4 | 49.7 | 103.3 | 49.7 | 103.3 | 21.5 | 29.8 | 37.2 | 25.7 | 42.3 | 37.6 |
| Gd ug/kg | 70.3 | 51.3 | 99.0 | 51.3 | 99.0 | 182.7 | 120.9 | 323.1 | 120.9 | 323.1 | 199.0 | 163.1 | 478.5 | 163.1 | 478.5 | 75.2 | 115.5 | 166.4 | 110.9 | 182.1 | 115.5 |
| Dy ug/kg | 39.2 | 29.6 | 56.3 | 29.6 | 56.3 | 111.4 | 65.9 | 182.5 | 65.9 | 182.5 | 90.6 | 87.2 | 247.3 | 87.2 | 247.3 | 47.2 | 62.8 | 78.3 | 69.0 | 96.5 | 78.2 |
| Ho ug/kg | 7.5 | 5.2 | 10.1 | 5.2 | 10.1 | 18.8 | 11.1 | 30.7 | 11.1 | 30.7 | 16.2 | 15.6 | 46.1 | 15.6 | 46.1 | 8.0 | 10.9 | 14.0 | 12.6 | 17.9 | 14.2 |
| Er ug/kg | 19.9 | 14.0 | 27.3 | 14.0 | 27.3 | 55.2 | 32.1 | 87.8 | 32.1 | 87.8 | 45.2 | 43.3 | 119.8 | 43.3 | 119.8 | 21.9 | 30.1 | 37.4 | 34.7 | 48.9 | 40.1 |
| Tm ug/kg | 1.80 | 1.23 | 2.43 | 1.23 | 2.43 | 5.47 | 2.97 | 5.82 | 2.97 | 5.82 | 4.26 | 3.74 | 10.10 | 3.74 | 10.10 | 2.03 | 2.67 | 3.12 | 3.32 | 4.27 | 3.95 |
| Yb ug/kg | 12.6 | 8.0 | 15.5 | 8.0 | 15.5 | 44.6 | 23.7 | 69.6 | 23.7 | 69.6 | 29.7 | 28.2 | 64.7 | 28.2 | 64.7 | 16.1 | 21.0 | 21.3 | 25.3 | 31.2 | 32.5 |
| Re ug/kg | 2.6 | 1.8 | 2.8 | 1.8 | 2.8 | 1.2 | 0.5 | 4.8 | 0.5 | 4.8 | 0.3 | 0.2 | 1.7 | 0.2 | 1.7 | 6.4 | 1.8 | 1.5 | 3.8 | 6.5 | 1.4 |
| Hg ug/kg | 30.3 | 24.1 | 231.4 | 24.1 | 231.4 | 46.2 | 43.0 | 48.5 | 43.0 | 48.5 | 17.0 | 12.7 | 18.0 | 12.7 | 18.0 | 12.3 | 19.5 | 19.1 | 15.3 | 54.8 | 12.6 |
| Tl ug/kg | 9.9 | 8.3 | 13.3 | 8.3 | 13.3 | 20.8 | 17.7 | 20.9 | 17.7 | 20.9 | 46.9 | 38.1 | 121.5 | 38.1 | 121.5 | 16.5 | 133.3 | 41.9 | 25.9 | 84.2 | 22.2 |
| Pb ug/kg | 367 | 351 | 633 | 351 | 633 | 1170 | 1002 | 1307 | 1002 | 1307 | 245 | 241 | 359 | 241 | 359 | 320.4 | 370.5 | 359.5 | 233.4 | 1468.2 | 351.6 |
| Bi ug/kg | 2.3 | 1.8 | 4.1 | 1.8 | 4.1 | 8.7 | 7.8 | 9.5 | 7.8 | 9.5 | 1.8 | 1.3 | 3.5 | 1.3 | 3.5 | 3.2 | 5.9 | 3.0 | 1.8 | 35.5 | 3.3 |
| Th ug/kg | 49.8 | 27.6 | 50.8 | 27.6 | 50.8 | 336.3 | 160.5 | 603.1 | 160.5 | 603.1 | 158.2 | 121.4 | 416.4 | 121.4 | 416.4 | 133.7 | 170.2 | 102.6 | 167.3 | 124.5 | 174.8 |
| U ug/kg | 4.3 | 2.9 | 5.2 | 2.9 | 5.2 | 52.5 | 22.6 | 68.7 | 22.6 | 68.7 | 15.8 | 10.5 | 37.4 | 10.5 | 37.4 | 18.8 | 28.2 | 21.4 | 14.1 | 23.5 | 21.6 |

**Figure S1.** Distribution of isotopic values (expressed in δ ‰) and elemental composition (expressed in g or mg or μg/kg, depending on the element).

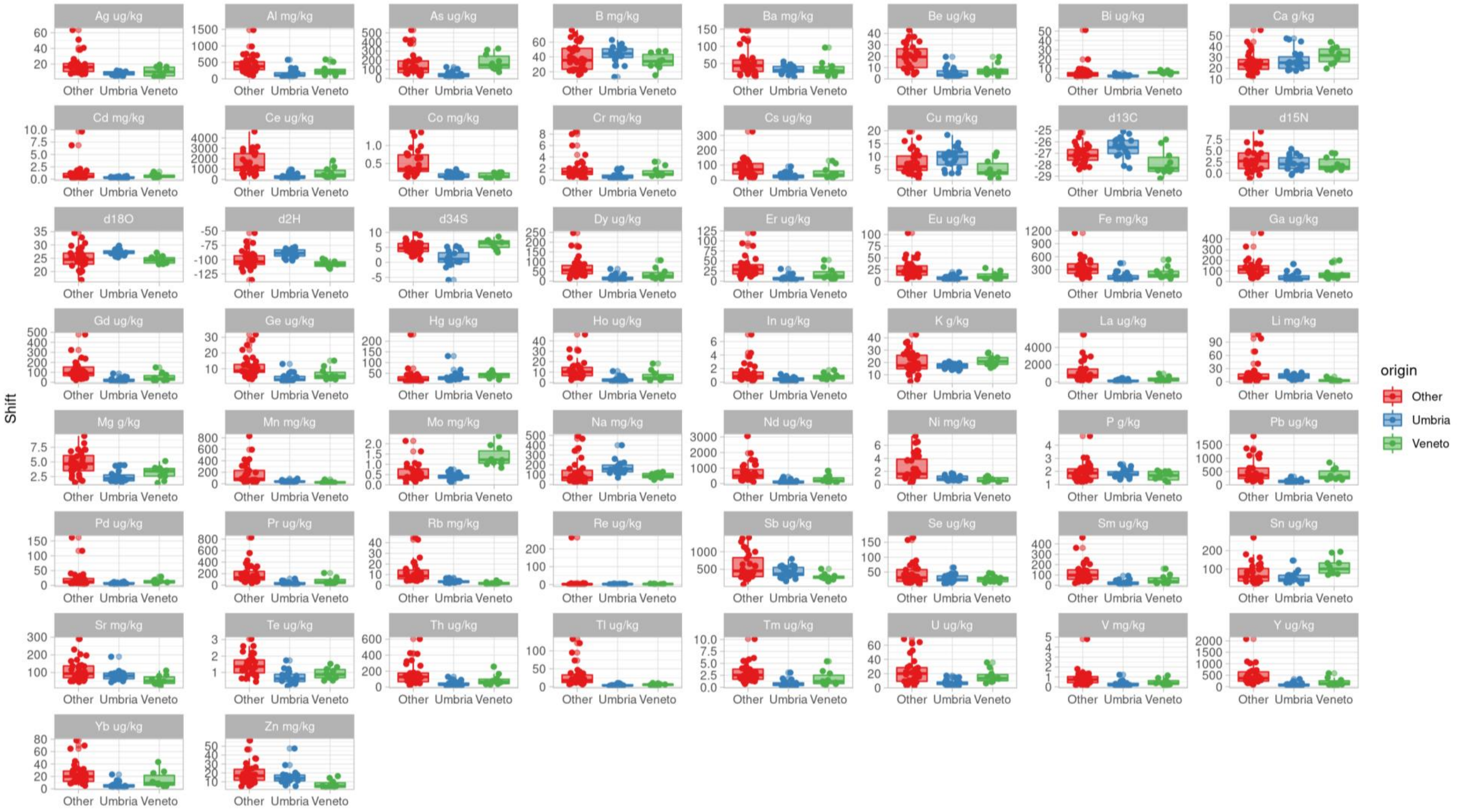

Supplement: Supplementary file 1 [file metabolites-11-00186-s001.pdf]
